# The Role of Behavioral Factors on Chronic Diseases—Practice and Knowledge Gaps

**DOI:** 10.3390/healthcare12242520

**Published:** 2024-12-12

**Authors:** Valentina Rahelić, Tomislav Perković, Lucija Romić, Pavo Perković, Sanja Klobučar, Eva Pavić, Dario Rahelić

**Affiliations:** 1 Department of Nutrition and Dietetics, University Hospital Centre Zagreb, 10000 Zagreb, Croatia; valentina.rahelic@kbc-zagreb.hr (V.R.); eva.pavic@kbc-zagreb.hr (E.P.); 2 Department of Dietetics, Nutrition and Analitycs Tehniqnes, University of Applied Health Sciences, 10000 Zagreb, Croatia; 3 Department of Food Technology, University North, 48000 Koprivnica, Croatia; 4 Vuk Vrhovac University Clinic for Diabetes, Endocrinology and Metabolic Diseases, Merkur University Hospital, 10000 Zagreb, Croatia; tomislavperkovic95@gmail.com (T.P.); lucijaromic@gmail.com (L.R.); 5 Department of Obstetrics and Gynecology, Merkur University Hospital, 10000 Zagreb, Croatia; pavo.perkovic@zg.t-com.hr; 6 Department of Internal Medicine, Division of Endocrinology, Diabetes and Metabolic Diseases, Clinical Hospital Centre Rijeka, 51000 Rijeka, Croatia; sanjaklobucarm@gmail.com; 7 Department of Internal Medicine, Faculty of Medicine, University of Rijeka, 51000 Rijeka, Croatia; 8 School of Medicine, Catholic University of Croatia, 10000 Zagreb, Croatia; 9 School of Medicine, Josip Juraj Strossmayer University of Osijek, 31000 Osijek, Croatia

**Keywords:** lifestyle medicine, chronic diseases, public health interventions, morbidity, mortality, disease prevention

## Abstract

**Background:** Behavioral factors, such as smoking, alcohol consumption, stress, poor diet, and physical inactivity, but also sleep deprivation and negative social connections, play a critical role in the development and progression of major chronic diseases. These include cardiovascular diseases, diabetes, chronic respiratory conditions, and cancers. **Methods**: The objective of this review is to explore the influence of these modifiable risk factors on the global burden of chronic diseases and assess the potential impact of public health interventions and policy changes. **Results:** The evidence highlights a significant association between behavioral risk factors and increased morbidity and mortality from chronic diseases. Public health interventions and policy changes targeting these modifiable behaviors have shown substantial potential in reducing the prevalence and impact of chronic conditions. Strategies such as smoking cessation programs, dietary improvements, physical activity promotion, and stress reduction are critical in mitigating these risks. **Conclusions:** Addressing modifiable behavioral factors is essential for the prevention and control of chronic diseases. Bridging the gap between current knowledge and effective implementation of interventions is crucial for improving population health outcomes. Public health strategies focused on modifying key behavioral risks can significantly reduce the burden of chronic diseases, thereby improving overall health and reducing healthcare costs.

## 1. Introduction

The global burden of chronic diseases has significant implications for global healthcare systems and economies, posing a worldwide challenge that requires prioritized prevention and management efforts. Chronic diseases are today the leading causes of mortality, with 41 million deaths each year and projected to rise to 52 million deaths and cost estimations reaching USD 47 trillion by 2030 [1]. Cardiovascular disease (CVD), diabetes, cancer, chronic lung illnesses, and mental health conditions are one of the leading global health concerns, largely driven by modifiable behavioral factors.

The burden of CVDs has nearly doubled, from 271 million cases in 1990 to 523 million in 2019 and it continues to be primary contributor to the global disease burden. For nearly all countries outside of high-income regions, the burden of CVD has steadily increased over the past several decades [2]. In 2021, an estimated 537 million people worldwide are living with diabetes, a number expected to rise to 643 million by 2030 and 783 million by 2045. Furthermore, around 541 million people are estimated to have impaired glucose tolerance as of 2021. Diabetes-related causes are projected to result in over 6.7 million deaths in 2021. Current direct healthcare expenditures due to diabetes are nearing USD 1 trillion and are anticipated to surpass this amount by 2030 [3]. By 2050, new cancer cases are projected to reach over 35 million, signifying a 77% increase from 2022. In 2022, an estimated 20 million new cancer cases and 9.7 million cancer-related deaths occurred globally. Currently, about 1 in 5 people will develop cancer in their lifetime, with approximately 1 in 12 women and 1 in 9 men dying from the disease [4]. It is estimated that between 2020 and 2050, cancers will cost the world economy USD 25.2 trillion [5]. According to the Global Burden of Diseases Study (GBD) 2017, chronic respiratory diseases like COPD and asthma remain significant contributors to global morbidity and mortality. Chronic obstructive pulmonary disease (COPD) was responsible for 3.2 million deaths, while asthma accounted for 495,000 deaths. COPD was the seventh leading cause of years of life lost (YLLs) [6]. Globally, there were 545 million cases of chronic respiratory diseases, with COPD and asthma each comprising approximately half. Each year, 62 million new cases are reported, primarily due to asthma (69%) and COPD (29%). COPD also contributed 81.6 million disability-adjusted life years (DALYs), while asthma added 22.8 million. By 2040, projections suggest that COPD will rise to become the fourth leading cause of YLLs, driven by population growth and aging [6]. Mental health conditions represent a significant and growing aspect of the global chronic disease burden, affecting over 970 million people worldwide. Depression and anxiety disorders are the most prevalent, with approximately 280 million individuals affected by depression and 301 million by anxiety disorders as of 2019 [7]. These conditions contribute substantially to disability-adjusted life years (DALYs), accounting for 14% of the global burden of disease. Furthermore, the economic impact of mental health disorders is projected to reach USD 16 trillion by 2030, largely due to lost productivity and healthcare costs [7,8].

While global trends highlight the rising burden of non-communicable diseases (NCDs), Europe faces its own unique challenges shaped by demographic shifts, lifestyle factors, and healthcare systems. Cardiovascular diseases alone account for up to 7.6% of total healthcare spending in the European Union (EU), while cancer contributes an additional 4–6%. Chronic respiratory diseases, including asthma, COPD, and lung cancer, add roughly 3.5% to total healthcare expenditures. Diabetes, particularly type 2 diabetes, shows cost variations across EU countries, ranging from 1.9% to 5.7% of total health expenditures. Combined, these four major NCDs impose direct costs equivalent to approximately 25.7% of the EU’s healthcare budget, with some countries like Germany and the Netherlands reporting higher percentages (28.5–34.3%). Indirect costs, stemming primarily from lost productivity due to these diseases, are estimated to range from 1.74% to 1.94% of the EU’s total GDP. Inpatient care constitutes the largest share of direct costs, consuming up to 12% of total healthcare spending. Despite these substantial figures, the estimates may be conservative, as they exclude private healthcare costs, non-medical expenses, and intangible costs like the value of lost life. Mental health conditions alone accounted for EUR 242 billion in direct costs and EUR 272 billion in productivity losses in the EU as of 2013, further highlighting the comprehensive impact of NCDs [9,10].

The rapidly growing global burden of chronic diseases reflects both population ageing and growth, as well as changes to people’s exposure to risk factors. Smoking, excessive alcohol consumption, poor diet, physical inactivity, and chronic stress are well-established contributors to these conditions [11]. Despite widespread awareness of these risk factors, the incidence of chronic diseases continues to rise, suggesting significant gaps between knowledge and practice in both the public and clinical realms. Understanding these behavioral drivers is crucial for effective prevention and management strategies, yet many healthcare systems still focus on treating symptoms rather than implementing comprehensive strategies to modify these behaviors. This reactive approach neglects opportunities for prevention and long-term management by failing to address the foundational lifestyle factors that drive chronic disease. Research has consistently demonstrated the impact of these behaviors on chronic disease outcomes. For example, smoking and poor diet are linked to cardiovascular disease, while physical inactivity and chronic stress increase the risk of metabolic disorders. However, despite the strength of the evidence, there are notable gaps in healthcare practice. One significant barrier is the gap between evidence-based recommendations and the practical realities faced by health professionals and individuals. For health professionals, limited time during consultations, inadequate training in behavior change strategies, and insufficient integration of multidisciplinary approaches hinder the promotion of lifestyle interventions. On the population level, socioeconomic factors such as financial constraints, limited access to healthy food, and safe spaces for physical activity pose significant obstacles. Additionally, cultural beliefs, low health literacy, and competing priorities in daily life often lead to a lack of adherence to health-promoting behaviors. The complexity of chronic disease management and the tendency to prioritize pharmacological treatments over behavioral interventions further complicate the issue. Bridging these gaps requires targeted education, systemic policy support, and accessible resources that address both structural and individual-level barriers [12,13,14,15].

## 2. Lifestyle Medicine

Lifestyle medicine is a growing field of healthcare that emphasizes addressing the root causes of chronic diseases, which are often linked to lifestyle choices. It focuses on six key pillars: whole-food, plant-predominant nutrition, physical activity, restorative sleep, stress management, avoidance of risky substances, and fostering positive social connections [16]. These elements, supported by substantial evidence, are instrumental in preventing and even reversing conditions like heart disease, diabetes, and obesity [17]. Recent studies underscore the economic and health benefits of lifestyle interventions. For instance, healthcare spending in the U.S. is heavily impacted by chronic diseases related to lifestyle, accounting for nearly 90% of healthcare costs [18]. Lifestyle medicine provides a cost-effective alternative by improving health outcomes through preventive measures. Research has shown that intensive lifestyle interventions can halt, and in some cases reverse, the progression of chronic diseases [19]. Dr. Dean Ornish’s program, which was the first lifestyle-focused cardiac rehabilitation program covered by Medicare, demonstrated significant improvements in heart health through lifestyle changes alone. Moreover, lifestyle medicine’s comprehensive approach aligns with the “quintuple aim” of healthcare: improving population health, enhancing patient care, reducing costs, supporting clinician well-being, and promoting health equity [20]. By equipping patients with the tools to manage their health proactively, lifestyle medicine holds promise for addressing health disparities that are deeply influenced by socioeconomic factors. Programs that integrate lifestyle medicine into community-based and healthcare settings can be particularly effective, offering resources to support sustainable behavior change. Thus, lifestyle medicine presents an evidence-based, preventive framework for managing chronic diseases and enhancing quality of life through behavior modification and environmental support [21].

## 3. Behavioral Factors

Behavioral factors play a significant role in determining health outcomes, affecting both the prevention and management of diseases. Key behaviors such as smoking, physical activity, diet, and alcohol use have been consistently linked to the risk of chronic diseases, including diabetes, heart disease, and certain cancers [22]. Behavioral science highlights that psychological constructs, such as self-efficacy and self-control, are critical for motivating and sustaining health behaviors, with frameworks like Social Cognitive Theory and the Transtheoretical Model providing tools for behavior modification interventions that emphasize goal-setting and self-monitoring [23]. For example, Social Cognitive Theory suggests that individuals’ confidence in their ability to make healthy changes, or self-efficacy, significantly impacts their health behaviors. This concept is reinforced by observational learning, where individuals model behaviors that are observed in their social environment [24]. Additionally, community and organizational support, as suggested by the Social Ecological Model, are necessary for reinforcing positive health choices and overcoming barriers like lack of access to resources or peer support, which impact behavioral health outcomes [5,23]. Barriers within organizations and communities include financial constraints, lack of integration between healthcare systems and community resources, and inadequate training or tools for health professionals [25]. For example, community health worker (CHW) programs, despite their potential to enhance health equity, often face issues such as insufficient funding and organizational resistance to adopting evidence-based approaches [25,26]. Additionally, communities may encounter systemic obstacles like limited access to nutritious food or safe spaces for physical activity [26]. Public health policies can act as enablers by fostering environments that support healthy choices. Strategies like implementing urban planning for walkable neighborhoods, subsidizing healthy food options, and mandating workplace wellness programs are effective [27]. Policies encouraging collaborations between health systems and community organizations are particularly impactful, promoting shared resources and improved access to care. Access to adequate resources, including education, infrastructure, and funding, is vital for sustained behavioral change [27,28]. Communities equipped with health-promoting resources—like recreation centers and mental health services—see better outcomes. The World Health Organization (WHO) emphasizes that tailored health programs, shaped by behavioral and cultural insights, can bridge gaps in community health initiatives [26]. A multi-level approach combining behavioral interventions, organizational change, community engagement, and supportive public policies can address these barriers effectively. Strengthening collaborations across sectors and investing in sustainable funding for community programs are key strategies for fostering healthier behaviors [26,28]. Health behaviors also impact aging, with research indicating that staying socially active, physically engaged, and mentally stimulated can enhance longevity and reduce the risk of cognitive decline. Behavioral and social factors are particularly impactful in later life, as they interact with biological and environmental determinants of aging [29]. Programs that support lifelong positive health behaviors, like exercise and balanced nutrition, play an essential role in maintaining cognitive and emotional health as individuals age. By emphasizing strategies that integrate psychological, social, and environmental support, health interventions can effectively target behavioral factors to improve long-term health outcomes across age groups [30].

### 3.1. Smoking

Smoking is one of the main environmental risk factors for various chronic diseases such as cardiovascular disease, arterial hypertension, type 2 diabetes, chronic obstructive pulmonary disease, and lung cancer. As far as CVDs are concerned, the trigger for the formation of atherosclerotic plaque is endothelial dysfunction caused by oxidative stress, i.e., free radicals released by cigarette smoke [31]. Endothelial dysfunction itself acts prothrombogenically; however, smoking also acts directly on thrombus formation by activating prothrombogenic factors and impairing fibrinolysis. Smoking also affects the lipid profile by increasing the concentration of total cholesterol, LDL-C, and triglycerides in the blood, which further worsens endothelial function. In addition, cigarette smoke causes oxidation of LDL-C, leading to the formation of foam cells that participate in the formation of atherosclerotic plaque [32]. It is believed that the pathophysiological mechanisms of arterial hypertension caused by smoking are an increase in sympathetic nerves and thus an increase in heart rate and contractility. On the other hand, a decrease in vasodilation due to a decrease in nitric oxide production causes increased arterial stiffness [33]. As for T2D, research has shown that cigarette smoke releases free radicals that damage pancreatic beta cells. On the other hand, apart from free radicals, epigenetic changes such as DNA methylation are also considered to be the cause of insulin resistance [34]. Smoking is the main cause of chronic obstructive pulmonary disease, emphysema, and lung cancer. The risk of developing these diseases increases with years of smoking experience, as well as with the number of smoked cigarettes per day. With long-term smoking, toxins and carcinogenic substances accumulate in the lower part of the respiratory tract and cause lung damage. Some of them are acrolein, formaldehyde, nitrogen oxides, polycyclic aromatic hydrocarbons, cadmium, hydrogen cyanide, and carbon monoxide [35]. Although nicotine does not directly affect carcinogenesis, by activating nicotinic acetylcholine receptors it can lead to the proliferation of cancer cells [36]. Cigarette smoke toxins activate macrophages that attract inflammatory cells that, together with macrophages, release proteases such as matrix metalloproteinase, causing elastin damage that leads to emphysema. In addition, macrophages stimulate the proliferation of fibroblasts leading to lung fibrosis, and mucus hypersecretion occurs through the release of neutrophil elastase [37]. Continuous exposure to cigarette smoke can lead to epithelial dysplasia and carcinogenesis by mutation of genes such as MYC, BCL2, and p53, which lead to small cell lung cancer. As for non-small cell lung cancer, there are genetic mutations in epidermal growth factor receptor, KRAS, and anaplastic lymphoma kinase [38,39]. The single most effective approach to slow down the progression of chronic obstructive pulmonary disease and to cut mortality by up to 50% is to stop smoking [40]. Evidence-based interventions, such as programs combining Cognitive-Behavioral Therapy (CBT) and Nicotine Replacement Therapy (NRT), have been shown to enhance quit rates effectively [41]. CBT is a structured, skills-based intervention that helps individuals recognize and modify thought patterns and behaviors associated with smoking. It includes strategies such as coping mechanisms, stress management, and relapse prevention [42]. Studies show that CBT, when combined with NRT, is more effective than CBT or NRT alone, yielding a relative risk of 1.53 for improved smoking abstinence over other combinations [42,43]. NRT delivers controlled amounts of nicotine without harmful tobacco combustion byproducts, alleviating withdrawal symptoms and reducing cravings. Available forms include patches, gums, lozenges, and inhalers. Combining long-acting (patch) with short-acting (gum or lozenge) NRT often increases the chances of quitting compared to single forms [41]. Programs integrating CBT and NRT capitalize on their complementary benefits. CBT addresses psychological and behavioral aspects, while NRT mitigates physiological dependence. For instance, a systematic review highlighted that such combination therapies produce higher quit rates compared to pharmacotherapy alone (relative risk of 1.39) [43]. Public health settings incorporating these methods via clinician-guided programs, group therapy, or digital platforms (e.g., text-based quitlines) have proven especially effective [41,44]. Despite their efficacy, challenges like access disparities, adherence issues, and variability in program implementation persist. Tailored interventions, enhanced community resources, and policy support to expand affordable cessation services are essential for broader impact. However, the problem is that even after such methods, studies show that most patients, up to 80% of them, continue to smoke one year after quitting [45]. The practice gaps related to smoking management are detailed in Table 1.

### 3.2. Alcohol

Alcohol has been a part of human culture for centuries, often associated with socialization, celebration, and relaxation. However, as we delve deeper into its effects on health, a growing body of evidence highlights the significant relationship between alcohol consumption and the development of chronic diseases [46]. As we navigate the intricate crossroads of health and societal habits, it is imperative to acknowledge the dual nature of alcohol’s impact on health. Alcohol consumption exhibits a dose-response relationship with chronic diseases, demonstrating varied effects at different intake levels. Moderate consumption (up to one drink per day for women and two for men) may confer a lower risk of certain cardiovascular diseases due to improved lipid profiles and insulin sensitivity [47]. Alcohol, particularly ethanol, stimulates an increase in high-density lipoprotein (HDL) cholesterol, often called “good cholesterol”. HDL is essential for reverse cholesterol transport, a process that helps remove cholesterol from arteries, reducing cardiovascular risk [48]. Ethanol in moderate quantities affects insulin signaling pathways by improving the function of insulin-sensitive tissues such as the liver and skeletal muscle. This improvement is likely due to a decrease in inflammatory markers and oxidative stress that impair insulin action [49]. Moderate alcohol intake has also been linked to reduced levels of low-density lipoprotein (LDL) cholesterol oxidation, which plays a key role in atherosclerosis development. The antioxidant properties of certain alcohol types, such as red wine, may contribute to this effect [50]. Moderate alcohol intake has been shown to increase adiponectin, a hormone secreted by adipose tissue that enhances insulin sensitivity and has anti-inflammatory properties. Elevated adiponectin levels help regulate glucose levels and reduce lipid accumulation in the liver, further supporting metabolic health [51]. However, this effect is controversial and potentially influenced by confounding factors like socioeconomic status and health behaviors [47,52]. Excessive alcohol use significantly elevates the risk of chronic conditions, including liver disease, hypertension, cancers, and cardiovascular complications, by promoting oxidative stress, inflammation, and organ damage [47,53]. Heavy drinking elevates blood pressure, disrupts lipid metabolism, and promotes the formation of arterial plaques, laying the foundation for conditions such as hypertension, atherosclerosis, and ultimately heart disease [54]. One of the most well-known associations with excessive alcohol consumption is liver disease. The pathophysiology of metabolic dysfunction-associated steatotic liver disease (MASLD) is a complex process involving various stages and mechanisms. Steatosis, characterized by the accumulation of fat in hepatocytes, marks the initial phase of MASLD. Alcohol disrupts the balance of lipid metabolism, leading to increased synthesis and decreased export of triglycerides. Progression to alcoholic hepatitis and cirrhosis occurs as inflammation and oxidative stress take their toll [55]. As MASLD advances, the liver’s ability to perform essential functions, such as detoxification, synthesis of proteins, and regulation of metabolism, becomes compromised. The loss of functional hepatocytes and the distorted architecture of cirrhotic liver tissue contribute to hepatic dysfunction. Individuals with cirrhosis due to MASLD are at an increased risk of developing hepatocellular carcinoma, a type of liver cancer [56]. Chronic inflammation and continuous regeneration of hepatocytes in response to liver injury create an environment conducive to the development of cancer. Globally, about 41% of all new diagnosed cancer cases in 2020 were linked to excessive alcohol intake [57]. The American Institute for Cancer Research identifies alcohol intake as the third leading modifiable risk factor for the development of cancer (after obesity and smoking). The International Agency for Research on Cancer categorizes alcohol as a Group 1 carcinogen, underscoring its role in cancer development [58]. Mechanistically, acetaldehyde, a metabolite of alcohol, induces DNA damage and impairs DNA repair mechanisms. Chronic alcohol exposure increases the risk of cancers affecting the mouth, throat, esophagus, liver, colon, rectum, and breast. The intricate interplay between genetic susceptibility and alcohol-induced carcinogenesis sheds light on the intricate relationship between cancer and alcohol intake [59]. Alcohol’s impact extends beyond the liver, affecting the nervous system and cognitive function. Chronic alcohol abuse leads to neurological disorders such as alcoholic neuropathy and Wernicke–Korsakoff syndrome. The neurotoxic effects of alcohol, coupled with nutrient deficiencies resulting from poor dietary habits often associated with heavy drinking, contribute to cognitive decline and motor impairments [60]. Alcohol’s influence on mental health is complex, involving both short-term intoxication effects and long-term consequences. While moderate alcohol consumption may have transient anxiolytic effects, chronic alcohol abuse is a well-established risk factor for mental health disorders. Depression and anxiety disorders are often exacerbated by heavy drinking, creating a cyclical pattern that poses significant challenges for both diagnosis and treatment [61]. Comprehensive public health guidelines emphasize the harms of overconsumption, urging caution with moderate drinking narratives [62]. Public health efforts to manage excessive alcohol consumption focus on strategies for prevention, screening, and intervention. Despite progress in addressing alcohol-related harm, significant shortcomings remain in the implementation of effective practices. Screening, Brief Intervention, and Referral to Treatment (SBIRT) is a widely recommended approach in healthcare. It involves screening patients for excessive alcohol use, providing counseling to reduce consumption, and referring individuals with Alcohol Use Disorder (AUD) to treatment. Research shows this method is underutilized; only about 11.6% of patients with AUD receive brief interventions during medical visits, and fewer are referred for treatment. This represents a critical gap in addressing AUD during routine care [63]. Measures like regulating alcohol outlet density, increasing taxes on alcohol, and enforcing dram shop liability laws are proven strategies to reduce alcohol availability and consumption. However, inconsistent enforcement and resistance from industry stakeholders limit their effectiveness. For example, stronger enforcement of laws prohibiting sales to minors has been recommended but remains inconsistently applied [64]. Electronic screening and brief intervention (e-SBI) tools have been shown to improve access to alcohol screening, particularly in non-clinical settings like workplaces and universities. However, their adoption is limited, partly due to a lack of training and awareness among providers [64]. Despite clear guidelines, screening for alcohol misuse remains inconsistent in healthcare. Barriers include time constraints, lack of provider training, and limited resources for follow-up interventions. Additionally, many clinicians are unaware of the best practices for addressing alcohol use disorders [65]. Patients often experience stigma and privacy concerns related to alcohol screening, which may reduce their willingness to disclose misuse. This underscores the need for nonjudgmental and supportive approaches during screening and counseling [65]. Fewer than 10% of individuals with AUD receive adequate treatment each year. This gap is exacerbated by shortages in addiction treatment services and insurance coverage disparities, which prevent many from accessing care [63]. Public health initiatives often focus on chronic heavy drinkers while neglecting early intervention opportunities for individuals at risk. This limits the effectiveness of preventive measures [64]. Addressing these challenges requires increased training for healthcare providers, expanded access to treatment, enhanced public awareness campaigns, and stronger regulatory measures to manage alcohol-related harm effectively. The practice gaps related to alcohol use management are detailed in Table 2.

### 3.3. Stress

In the fast-paced modern world, stress has become an omnipresent force, influencing not only mental well-being but also playing a pivotal role in the development and progression of chronic diseases. The body’s stress response, orchestrated by the hypothalamic-pituitary-adrenal axis and the sympathetic nervous system, is a crucial survival mechanism. However, chronic activation of these systems in response to persistent stressors can lead to dysregulation, contributing to the onset of chronic diseases [66]. Chronic stress has been linked to a pro-inflammatory state within the body. The release of stress hormones, such as cortisol, activates immune cells and promotes the synthesis of inflammatory molecules. Prolonged inflammation is a hallmark of various chronic diseases, including CVDs, diabetes, and autoimmune disorders [67]. Epidemiological studies consistently highlight the association between chronic stress and an increased risk of CVD. Prolonged stress stimulates the sympathetic nervous system, leading to increased secretion of catecholamines like norepinephrine and epinephrine. This can cause vascular remodeling, increase peripheral resistance, and raise blood pressure, contributing to hypertension. Additionally, it induces oxidative stress and endothelial dysfunction, further exacerbating cardiovascular risks [68]. Stress can activate platelets and enhance the coagulation system, contributing to a pro-thrombotic state. Increased platelet activation and coagulation can elevate the risk of thrombus formation, leading to conditions such as myocardial infarction and stroke [69]. Chronic stress has profound effects on metabolism, potentially leading to insulin resistance and the development of T2D. Stress-induced cortisol release can disrupt glucose homeostasis, contributing to elevated blood sugar levels. Chronic exposure to elevated cortisol levels can lead to insulin resistance, reducing the responsiveness of the body’s cells to insulin [70]. Insulin resistance is a hallmark of T2D, as it impairs the ability of cells to efficiently absorb glucose from the bloodstream. Chronic low-grade inflammation is related to insulin resistance and contributes to the progression of T2D. Inflammatory cytokines such as tumor necrosis factor-α and interleukin-6 interfere with insulin signaling pathways, further exacerbating insulin resistance and impairing glucose uptake by cells [71]. Excessive cortisol enhances hepatic gluconeogenesis and suppresses peripheral glucose uptake by inhibiting insulin signaling in tissues such as muscle and adipose tissue [72]. Moreover, stress-related behaviors, such as overeating and poor dietary choices, play a role in the development of obesity and metabolic syndrome [73]. The interplay between stress and mental health is bidirectional, with chronic stress contributing to the development of mental health disorders and pre-existing mental health conditions intensifying the body’s stress response. Stress is a well-established risk factor for mood disorders, anxiety, and depression, conditions that, in turn, are linked to an increased risk of chronic diseases [74]. Stress-induced alterations in the immune system compromise the body’s ability to defend against infections and maintain immune balance. Chronic stress has been associated with increased susceptibility to infections, delayed wound healing, and a higher risk of autoimmune diseases [75]. Chronic stress elevates pro-inflammatory cytokines such as interleukin-6 (IL-6) and tumor necrosis factor-alpha (TNF-α), which contribute to systemic inflammation. This low-grade inflammatory state plays a role in the progression of autoimmune diseases [75,76]. Stress-induced immune changes also suppress adaptive immunity, which can increase susceptibility to infections while exacerbating autoimmune responses. Stress is linked to increased disease activity in autoimmune conditions like rheumatoid arthritis and lupus. By promoting a pro-inflammatory milieu and disrupting the balance between immune cell types, stress worsens tissue damage and autoimmunity. Additionally, HPA axis dysfunction can impair the body’s ability to regulate these immune responses [76]. Understanding stress as a driver of chronic diseases necessitates a holistic approach that considers the intricate interplay between molecular pathways, organ systems and psychological well-being. Strategies for managing stress, such as mindfulness, exercise, and social support, can mitigate the negative health effects associated with chronic stress. By recognizing stress as a modifiable risk factor, healthcare professionals and individuals alike can work towards promoting health resilience and preventing the cascade of events that lead to chronic diseases [77]. The practice gaps related to stress management are detailed in Table 3.

### 3.4. Ultra-Processed Foods

There is strong evidence that there is association between dietary habits and the prevention and treatment of obesity, T2D, CVDs, and different types of cancer [78]. One of the dietary issues of modern society is a high intake of ultra-processed foods, which results in increased energy intake and weight gain, and therefore has a negative impact on health [79], especially on cardiovascular, coronary heart, and cerebrovascular diseases [80]. The more processed food is, the higher the glycaemic response and the lower its satiety; this can influence physiology [81], lead to overeating, and consequently bring about metabolic disorders [82]. Most often, this food also contains high amounts of sodium, sugar, and unhealthy types of fat [81] and contains low amounts of fibers, so it should be fortified to include them in the form of functional fibers or whole grains [83]. The SUN Spanish cohort study reported that the highest risk for T2D was found in those with the highest consumption of ultra-processed foods [84], and a multinational cohort study concluded that higher consumption (260 g per day) is associated with a higher risk of developing a combination of chronic diseases, including cardiometabolic multimorbidity (T2D and CVD) and cancer. Significant associations have been observed with artificially sweetened beverages, sugar-sweetened beverages, and products of animal origin [85]. Mechanisms on how ultra-processed food may influence the development of chronic diseases and multimorbidity are still not completely understood. It is considered that different factors in processing might have a role in this association, such as final product nutritional composition, contact materials, additives, and neoformed contaminants, but further studies are needed to understand relative endowments [80]. Ultra-processed food (UPF) is a source of at least half of the amount of daily salt intake. It is known that there is a positive association between high salt intake and high blood pressure, which can result in negative cardiovascular events [86]. The WHO daily intake goal for adult population is 5 g of salt (200 mg sodium) [87], so for effective salt intake reduction, it is important that consumers are aware of product information, nutrition facts, and that the food industry actively participates in different public health actions and campaigns [86]. Such food also often has a longer shelf life due to the use of different preservatives and it is longer exposed to effects of various chemicals such as bisphenol A and phthalates, which are known to have a disruptive influence on the endocrine system; they are known as endocrine disruptors, and are associated with the development T2D [88]. Physical and chemical processes during production (such as heating at high temperatures) can lead to the formation of contaminants that may have a health risk; for example, acrylamide present in fried potatoes, biscuits, crackers, and coffee is associated with insulin resistance. Other processes, such as hydrogenation, pre-frying, shaping, extrusion, etc., can also lead to the production of various compounds with potentially harmful effects on health, and ultra-processed food usually goes through a greater number of these processes [89]. Furthermore, it usually contains some components that are not so often used in everyday food preparation, components such as refined sugars, hydrogenated oils, and various additives, and for some of these components, cardiometabolic effects have already been determined [80]. UPFs such as sugary drinks, packaged snacks, instant noodles, and processed baked goods often contain refined carbohydrates and added sugars, causing rapid spikes in blood glucose levels. This hyperglycemia triggers excessive insulin release, promoting insulin resistance over time. Chronic hyperinsulinemia leads to weight gain, systemic inflammation, and dyslipidemia, all hallmarks of metabolic syndrome. Studies link high UPF consumption to an increased risk of type 2 diabetes due to impaired glucose tolerance [90]. Additives such as emulsifiers and preservatives alter gut microbiota composition, leading to dysbiosis. This imbalance increases intestinal permeability, allowing bacterial endotoxins to enter circulation and trigger low-grade chronic inflammation. Persistent inflammation exacerbates insulin resistance and contributes to atherosclerosis, a precursor to cardiovascular diseases [91]. Many UPFs such as vegetable shortenings, cookies, pastries, chips, crackers, fried chicken, french fries, frozen pizzas, and other pre-prepared meals contain hydrogenated oils rich in trans fats, which elevate LDL cholesterol and decrease HDL cholesterol. This lipid imbalance promotes plaque formation in arteries, raising the risk of hypertension, myocardial infarction, and stroke. A diet high in UPFs has been associated with higher levels of circulating triglycerides and C-reactive protein, a marker of inflammation [92]. Also, UPFs contain advanced glycation end products (AGEs), formed during high-temperature cooking and processing (grilled, roasted, or fried meats, packaged baked goods, sweetened condensed milk and processed cheeses, potato chips, puffed snacks, and sweetened beverages). AGEs generate reactive oxygen species (ROS), leading to oxidative damage to cells and tissues. Oxidative stress further drives inflammation and endothelial dysfunction, which are critical in the pathogenesis of cardiovascular diseases [93]. In conclusion, it is clear that consumption of minimally processed or unprocessed foods, by reducing energy density and increasing satiety, can affect weight management and improve appetite control. Therefore, it is necessary to consider how to limit consumption and advertising of processed food and promote well established dietary patterns with fresh food and minimally processed food [79,81]. The practice gaps related to poor diet management are detailed in Table 4.

### 3.5. Low Fruit and Vegetable Intake

Fruit and vegetables are a significant part of healthy dietary patterns. They are important sources of different nutrients and phytochemicals and are low in sodium and fat but high in potassium and fiber [94]. According to the EAT-Lancet reference diet recommendations, daily intake should be 200 g of fruits with a range of 100–300 g and 300 g of vegetables with a range of 200–600 g [95], and the WHO recommendation is that minimum intake of fruits and vegetables is 400 g per day [96]. It is well known that increased fruit and vegetable intake, five servings per day, can have positive influence on prevention of coronary heart disease, stroke, and hypertension, and may aid in weight management and thus reduce risk of development of T2D. They also decrease the risk of chronic obstructive pulmonary disease, asthma, osteoporosis, rheumatoid arthritis, and dementia [97,98,99]. Studies have shown that some fruits and vegetables, especially garlic and cruciferous vegetables, contain antioxidants that protect cells from oxidative stress and DNA from damage [100,101], consequently lowering the incidence of cancer, but evidence on cancer outcomes remains limited [102]. Compounds such as quercetin (found in apples and onions) and curcumin (from turmeric) inhibit inflammatory pathways by suppressing pro-inflammatory cytokines and enzymes like cyclooxygenase-2 (COX-2). This mechanism is significant in managing diseases like diabetes and arthritis, which are driven by chronic inflammation [103]. Phytonutrients interact with gut microbiota, enhancing the production of beneficial metabolites like short-chain fatty acids (SCFAs). Polyphenols from foods such as green tea and grapes have been shown to promote the growth of beneficial bacteria while suppressing pathogenic strains. This modulation improves gut health and systemic immune responses, reducing the risk of metabolic disorders [104,105]. Flavonoids like catechins (in tea) and resveratrol (in grapes) improve endothelial function, reduce LDL cholesterol oxidation, and enhance nitric oxide availability, which collectively lower the risk of atherosclerosis and hypertension [105]. Phytonutrients influence key metabolic pathways. For instance, sulforaphane in cruciferous vegetables upregulates antioxidant defenses and detoxification enzymes, helping to mitigate insulin resistance and other metabolic dysfunctions associated with type 2 diabetes [104,105]. Recent studies also found that there is significant inverse association between fruit and vegetable consumption and the risk of inflammatory bowel diseases [106]. In conclusion, within the evidence base, it is clear that higher intakes of fruit and vegetables (five servings per day) can have positive effect on the prevention of chronic diseases. Despite this, recommended daily intake is not fulfilled around the world. It is important to develop effective methods and tools for education and dissemination of information in order to adopt adequate eating patterns from childhood that are in line with recommendations. It is also important to emphasize consumption of seasonal and locally grown food as it has more benefits on nutrition, the local economy, and the environment [107,108]. The practice gaps related to poor diet management are detailed in Table 4.

### 3.6. Low Physical Activity

Low physical activity levels are associated with increased risk for chronic disease development, with the most pronounced being ischemic heart disease (23%), T2D (44%), and certain types of cancer (7 to 41%) [109]. It is known that individuals that are inactive have between 150% and 240% greater risk for development of CVDs in contrast to those that are regularly active [110]. Regular activity can have a positive influence on metabolic dysregulation, prevention of obesity, and thus the development of different cancers such as esophageal, gallbladder, pancreatic, liver, colorectal, kidney, uterine, and breast cancer [111]. Increased and regular physical activity can influence fatty acid oxidation and can influence blood sugar regulation; therefore, it is an important tool in prevention of metabolic disease. In addition, moderate and regular exercise improves the metabolic phenotype of adipose tissue, skeletal muscle, liver, and pancreas and increases the ability of skeletal muscle to absorb glucose [112]. This process is crucial for managing blood sugar levels, especially for individuals at risk of type 2 diabetes. Exercise enhances insulin sensitivity through various mechanisms, including the increased expression of glucose transporters on muscle cells and the activation of signaling pathways like AMP-activated protein kinase (AMPK), which regulates energy balance [113]. Additionally, exercise increases mitochondrial function in skeletal muscle, which improves energy production and fat oxidation, further reducing the risk of metabolic diseases [113,114]. Exercise also plays a key role in lipid metabolism. Regular aerobic activity helps decrease circulating triglyceride levels while increasing high-density lipoprotein (HDL) cholesterol, which is protective against cardiovascular diseases. At the same time, exercise reduces low-density lipoprotein (LDL) cholesterol, commonly associated with atherosclerosis. These lipid profile improvements are partly due to exercise-induced increases in lipoprotein lipase (LPL) activity, an enzyme that helps break down triglycerides in blood [115]. Furthermore, exercise induces the release of myokines—signaling proteins secreted by muscles—that promote inter-organ communication and influence processes in adipose tissue, liver, and other organs. These myokines help modulate inflammation and energy metabolism, reducing chronic low-grade inflammation, which is a key contributor to many metabolic and cardiovascular diseases [116]. The effects of exercise on the gut microbiome have also gained attention, with exercise promoting the growth of beneficial bacteria that produce metabolites associated with reduced inflammation and improved metabolic health [117]. In addition, it has many proven benefits on improving symptoms of anxiety, depression, and distress, which can influence sleep quality [118], reduces risk of dementia, and improves cognitive function [119]. Therefore, WHO recommends at least 150–300 min of moderate aerobic physical activity or 75–150 min of vigorous-intensity aerobic physical activity per week for adults. Additional recommendations include limiting the amount of sedentary of time and screen time, which can also lead to sedentary behavior [120,121]. One of the possible solutions to raise physical activity level is to increase levels of walkability by increasing the number of green spaces and parks, as it is known that there are positive associations between walkable park areas and lower rates of obesity and T2D [122]. It should be emphasised that regular physical activity, preferably 30 min daily, has positive outcomes in obesity and chronic disease prevention, mood improvements, and overall health, therefore improving overall quality of life. It should also be encouraged in those who already have some chronic diseases, such as T2D, hypertension, and/or osteoarthritis, as it can help reduce the risk of progression of the condition and development of complications related to their diagnosis [119]. The practice gaps related to physical inactivity management are detailed in Table 5.

### 3.7. Sleep Deprivation

Sleep deprivation is increasingly recognized as a significant contributor to chronic diseases such as obesity, anxiety, depression, and heart disease [123]. Despite extensive research, there remain critical gaps in both understanding and practical approaches to mitigating these risks. Sleep deprivation can result from various factors, including lifestyle choices such as prioritizing work, entertainment, or social activities over sleep [124]. Conditions such as insomnia, sleep apnea, and restless legs syndrome disrupt sleep. Anxiety and depression can interfere with the ability to fall or stay asleep [124,125]. Environmental factors such as noise, light pollution, or uncomfortable sleeping conditions also disrupt sleep. Shift work and irregular schedules disrupt the body’s circadian rhythm, making restorative sleep difficult [125]. Chronic pain or illnesses can also lead to fragmented or insufficient sleep [124,125]. Sleep deprivation disrupts the balance of hormones regulating hunger, such as ghrelin and leptin, leading to increased appetite and a preference for high-calorie foods [126]. Over time, this contributes to weight gain and obesity. However, many interventions focus on diet and exercise without adequately addressing sleep health, a crucial modifiable risk factor. Sleep deprivation significantly impacts glucose metabolism and insulin sensitivity [127]. Studies show that insufficient sleep increases insulin resistance, a key factor in developing type 2 diabetes [127]. Chronic sleep loss can lead to a “vicious cycle”, where poor metabolic control further disrupts sleep, worsening health outcomes [127,128]. Importantly, short sleep duration (less than 6 h per night) is strongly associated with a heightened risk of type 2 diabetes [128]. Sleep loss exacerbates anxiety and depression by impairing emotional regulation and increasing stress hormones like cortisol [123,129]. The bidirectional relationship between sleep and mental health remains understudied, with limited emphasis on sleep-focused interventions in mental health treatment plans [129,130]. Sleep plays a critical role in immune regulation. During sleep, the body produces and releases cytokines, proteins that combat inflammation and infection. Sleep deprivation reduces the production of these protective molecules, weakening the immune response. This leaves individuals more susceptible to infections, delays recovery, and potentially increases the risk of chronic inflammation-related diseases [131]. Insufficient sleep is linked to hypertension, atherosclerosis, and coronary artery disease. Chronic sleep deprivation hinders blood pressure regulation and promotes systemic inflammation, elevating CVD risks [132]. Despite this, public health initiatives often under-prioritize sleep education as a preventive measure. While associations between sleep deprivation and chronic diseases are well established, the mechanisms underlying these relationships are not fully understood. For example, how different sleep stages specifically affect metabolic and cardiovascular health requires further exploration [123,132]. Prevention strategies to combat sleep deprivation are to maintain a consistent sleep schedule (go to bed and wake up at the same time daily). It is important to create a sleep-friendly environment, ensuring that your bedroom is dark, quiet, and cool. Another helpful piece of advice to prevent sleep deprivation is to avoid caffeine and screens close to bedtime [124]. Also, excessive daytime napping can interfere with nighttime sleep. By addressing these factors, individuals can improve their sleep quality, thereby reducing the risks associated with sleep deprivation [124]. Despite all these strategies, practical solutions to improve sleep remain underdeveloped. Additionally, disparities in sleep access and quality among different socioeconomic groups are insufficiently addressed. There is a lack of widespread understanding about the critical role of sleep in preventing chronic diseases. Educational campaigns and accessible sleep health resources are essential, but are underfunded and overlooked [124,133]. Addressing these gaps requires integrated efforts from researchers, policymakers, healthcare providers, and educators. By prioritizing sleep health as a foundational component of chronic disease prevention, we can improve individual well-being and reduce healthcare costs. The practice gaps related to management of sleep deprivation are detailed in Table 6.

### 3.8. Negative Social Connections

Negative social connections, encompassing loneliness, social isolation, and toxic relationships, have profound implications for the development of chronic diseases such as obesity, anxiety, depression, and heart disease [134]. These relationships act as stressors that exacerbate physiological and psychological burdens, yet gaps in both practice and research hinder effective interventions. Negative social interactions activate the HPA axis, leading to prolonged elevation of cortisol [135]. Chronic hypercortisolemia can impair glucose metabolism, increase visceral fat deposition (contributing to obesity), and suppress hippocampal neurogenesis (associated with anxiety and depression) [135]. Social isolation is associated with elevated levels of pro-inflammatory cytokines, such as interleukin-6 (IL-6) and tumor necrosis factor-alpha (TNF-α). Chronic inflammation contributes to the pathogenesis of cardiovascular diseases (atherosclerosis) and metabolic syndrome, which are linked to obesity [135,136]. Negative social connections are also linked to increased oxidative stress through excessive production of reactive oxygen species (ROS), which damage cells and tissues. This contributes to aging, atherosclerosis, and neurodegenerative changes associated with chronic diseases. For instance, social isolation has been linked to a 29% increased risk of coronary heart disease and a 32% increased risk of stroke [136]. Similarly, loneliness is associated with higher rates of obesity, likely due to stress-induced overeating and sedentary behavior [137]. Psychological conditions such as anxiety and depression are also exacerbated by poor social connections, creating a feedback loop where mental health deterioration increases physical health risks [134]. Despite clear associations, understanding the interplay of social relationships with chronic diseases remains incomplete. For instance, most studies focus on either loneliness or social isolation without adequately exploring how these constructs differ in their effects. Additionally, cultural and socioeconomic factors influencing social dynamics and their health outcomes are understudied, leaving interventions potentially mismatched to diverse populations [138]. On the practical side, integrating social health into medical care is limited. Current healthcare systems rarely assess social connections systematically, despite their comparable impact on mortality risks related to smoking or obesity. Interventions are also fragmented, often treating psychological or physical conditions in isolation without addressing their social determinants. Furthermore, long-term strategies to mitigate the effects of poor social connections are lacking, particularly in vulnerable groups such as the elderly [138,139]. To bridge these gaps, comprehensive frameworks that include routine social health screenings and culturally tailored interventions are essential. Research should delve into the nuanced mechanisms by which negative social connections influence health and evaluate the effectiveness of integrative approaches [139]. The practice gaps related to management of negative social connections are detailed in Table 7.

## 4. Healthcare Policy: Portable Interventions and Policy Innovations

The increasing prevalence of lifestyle-related health issues necessitates innovative healthcare policies. Portable interventions—strategies that can be easily adapted and implemented across various settings—are essential in addressing these challenges. For smoking cessation, policies that promote mobile applications and telehealth services have shown promise. These platforms provide personalized support and resources to individuals seeking to quit smoking [140]. Similarly, for alcohol misuse, digital interventions like online counseling and self-monitoring apps can help individuals manage their consumption effectively [141]. Stress management is another critical area where portable interventions can make a significant impact. Mindfulness-based programs delivered through mobile devices have been effective in reducing stress levels among users [142]. Furthermore, promoting healthy dietary habits through mobile nutrition apps can assist individuals in making better food choices by providing real-time feedback on their dietary intake [143]. Lastly, encouraging physical activity through wearable fitness trackers has gained traction as a policy innovation. These devices not only monitor activity levels but also foster community engagement through social sharing features [144]. By integrating these portable interventions into healthcare policy frameworks, we can create a more holistic approach to public health that addresses the multifaceted nature of lifestyle-related diseases.

## 5. Conclusions

In this review, we explored the role of behavioral factors—smoking, alcohol consumption, stress, poor diet, physical inactivity, sleep deprivation, and negative social connections—on the development and progression of chronic diseases. Our findings align with a robust body of research indicating that these behaviors are major contributors to the global burden of non-communicable diseases (NCDs), including cardiovascular disease, diabetes, cancer, and chronic respiratory conditions [145]. However, we identified significant knowledge gaps in addressing these factors, both at the individual and systemic levels, as well as in translating knowledge into practical public health interventions. First, the interplay between behavioral factors and genetic predisposition remains an under-explored area. While previous studies have highlighted the influence of genetics on disease susceptibility, they often fail to comprehensively account for how behavioral modification can alter these risks. For example, genetic insights can help identify individuals with a heightened susceptibility to nicotine dependence or smoking-related illnesses, enabling tailored prevention strategies. Individuals with genetic variations influencing nicotine metabolism may benefit from targeted pharmacological treatments or specific cessation aids. Future research should prioritize examining these intersections to personalize prevention strategies [146]. Second, the review highlighted the importance of stress as a significant behavioral factor contributing to chronic diseases, such as hypertension, cardiovascular diseases, and mental health disorders. The literature establishes clear pathways linking chronic stress to physiological changes, such as increased cortisol levels, systemic inflammation, and immune dysfunction, which collectively exacerbate disease progression. While psychological interventions, such as mindfulness-based stress reduction (MBSR) and cognitive-behavioral therapy (CBT), have shown effectiveness in stress management, there is growing evidence that multi-faceted approaches, combining lifestyle changes, social support, and medical interventions, offer more comprehensive benefits.

## 6. Future Directions

However, there is still a need for further research to evaluate the long-term effectiveness of these combined approaches, especially in diverse populations and varying socio-economic contexts. Research could investigate whether combining pharmacological aids with community-driven support systems or stress management programs yields better outcomes in low-resource settings [147]. Moreover, poor diet and physical inactivity remain pervasive global issues, yet many studies fail to adequately address the socio-economic and environmental barriers that impede healthy behavior changes. Interventions designed to promote healthier lifestyles are often one-size-fits-all, disregarding the cultural, economic, and logistical challenges faced by disadvantaged groups. The practice gap here lies in the lack of tailored interventions that account for these social determinants of health [148]. Additionally, the review reveals that despite public health campaigns, smoking and excessive alcohol consumption persist as significant contributors to chronic diseases. This indicates a gap in the efficacy of behavior change communication strategies, suggesting that future research should explore more innovative and personalized approaches, including digital health tools and community-based initiatives [149]. In conclusion, while behavioral factors are well-established contributors to chronic disease, there remain substantial gaps in both our understanding and the application of interventions. Future research should aim to integrate genetic, psychosocial, and environmental factors into more personalized and culturally sensitive public health strategies to mitigate the global burden of NCDs.

## Figures and Tables

**Table 1:** Practice Gaps in Management of Smoking *.

Practice Gap	Description	Proposal to Close Gap
Limited integration of smoking cessation into routine care.	Smoking cessation is often overlooked during routine consultations, missing a critical opportunity for intervention.	Implement standardized smoking cessation protocols, including brief interventions, in routine patient visits across all specialties.
Insufficient training in motivational interviewing.	Healthcare providers may lack skills in motivational interviewing techniques that are effective in encouraging quitting.	Integrate motivational interviewing training into healthcare professional education and offer refresher courses for current staff.
Lack of follow-up support for individuals attempting to quit.	Many patients relapse due to lack of consistent follow-up and support throughout the quitting process.	Establish follow-up systems, such as check-ins or referrals to support groups, to maintain patient engagement post-intervention.

* Practice gaps are represented by inadequate translation of knowledge into action.

**Table 2:** Practice Gaps in Management of Alcohol Use *.

Practice Gap	Description	Proposal to Close Gap
Inadequate screening for alcohol use disorders.	Alcohol use is often under-assessed in primary and specialty care, leading to missed opportunities for early intervention.	Implement routine alcohol use screenings as part of standard assessments across healthcare settings.
Limited training in brief interventions.	Many healthcare providers lack training in delivering brief, evidence-based interventions to reduce harmful alcohol consumption.	Include brief intervention training in healthcare curricula and offer continuing education courses on alcohol intervention strategies.
Lack of multidisciplinary support for recovery.	Patients with alcohol dependence often need coordinated care, but access to integrated services (e.g., counseling, social support) is limited.	Develop collaborative care pathways that link primary care providers with mental health, social work, and addiction counseling services.

* Practice gaps are represented by inadequate translation of knowledge into action.

**Table 3:** Practice Gaps in Management of Stress *.

Practice Gap	Description	Proposal to Close Gap
Infrequent assessment of stress levels.	Stress is often not routinely assessed, despite its role in exacerbating many chronic conditions and mental health issues.	Integrate stress screening tools into routine healthcare assessments to identify high-risk individuals.
Limited provider training in stress management.	Healthcare providers may lack training in evidence-based stress management techniques, such as mindfulness or relaxation methods.	Incorporate stress management techniques into medical and continuing education programs.
Insufficient referral pathways for stress support.	Patients who report high stress often lack access to adequate support services, such as counseling or behavioral health resources.	Develop streamlined referral systems to connect patients with counseling, support groups, and behavioral health services as needed.

* Practice gaps are represented by inadequate translation of knowledge into action.

**Table 4:** Practice Gaps in Management of Poor Diet *.

Practice Gap	Description	Proposal to Close Gap
Inadequate nutritional screening in primary care.	Nutritional status and dietary habits are not consistently assessed during routine care, missing opportunities for early intervention.	Implement brief dietary assessment tools in primary care visits to identify dietary risks.
Limited provider training in dietary counseling.	Many healthcare providers lack sufficient training to offer effective dietary counseling or personalized nutrition advice.	Incorporate basic nutrition education and dietary counseling training in medical and nursing curricula, and offer workshops for current staff.
Poor access to multidisciplinary nutrition support.	Patients often lack access to dietitians or other nutrition specialists who can provide targeted dietary support and follow-up care.	Establish referral pathways to registered dietitians or nutritionists, and consider integrating dietitians into primary care teams where feasible.

* Practice gaps are represented by inadequate translation of knowledge into action.

**Table 5:** Practice Gaps in Management of Physical Inactivity *.

Practice Gap	Description	Proposal to Close Gap
Lack of routine assessment of physical activity.	Physical activity levels are rarely assessed during medical visits, limiting opportunities to identify and support inactive patients.	Integrate standardized physical activity questionnaires into routine assessments in primary care.
Insufficient training in exercise counseling.	Many healthcare providers feel underprepared to advise on exercise, lacking knowledge of evidence-based activity guidelines.	Offer training for healthcare providers on exercise counseling, focusing on practical guidance aligned with physical activity guidelines.
Limited access to exercise referral programs.	Patients often have few resources or structured programs to help them start and maintain physical activity routines.	Establish community-based exercise referral schemes and partnerships with fitness facilities to improve access to guided exercise programs.

* Practice gaps are represented by inadequate translation of knowledge into action.

**Table 6:** Practice Gaps in Management of Sleep deprivation *.

Practice Gap	Description	Proposal to Close Gap
Inadequate screening for sleep deprivation in primary care.	Many healthcare providers fail to screen for sleep disorders, or they may not recognize the early signs of sleep deprivation.	Integrate standardized questionnaires.
Insufficient integration of behavioral interventions for sleep management.	Many patients with sleep deprivation are simply prescribed medication or are left with generic advice on improving sleep hygiene.	Increase the availability of evidence-based behavioral treatments, through training programs for clinicians and expanding access to digital health platforms.
Limited public awareness of sleep’s role in chronic disease prevention.	Many individuals are unaware of the long-term health risks associated with insufficient sleep, such as an increased risk for obesity, diabetes, hypertension, and mental health disorders.	Public health campaigns should emphasize the importance of sleep for chronic disease prevention. Schools, workplaces, and healthcare organizations should collaborate to promote sleep hygiene and the benefits of adequate sleep.

* Practice gaps are represented by inadequate translation of knowledge into action.

**Table 7:** Practice Gaps in Management of Negative social connections *.

Practice Gap	Description	Proposal to Close Gap
Under recognition of the impact of social isolation in healthcare.	Healthcare professionals often overlook social isolation as a risk factor for health problems, focusing primarily on medical conditions or individual lifestyle factors.	Routine screening for social isolation. Healthcare providers should be trained to ask about social support during consultations and recognize how patients’ social environments influence their overall well-being.
Limited integration of social support interventions in mental health treatment.	While mental health interventions often focus on individual therapy and medication, the role of social support in recovery is frequently underestimated.	Family therapy, couples counseling, or social skills training to help patients build positive relationships. Implementation of programs that focus on improving social cohesion and communication skills.
Inadequate addressing of toxic relationships in chronic disease management.	Healthcare providers often fail to directly address toxic relationships during chronic disease treatment, focusing instead on medication and lifestyle changes without considering the emotional or social context.	Incorporation of emotional health assessments into regular follow-up appointments. Providers should offer resources like therapy or conflict resolution programs. Training healthcare professionals could enhance their ability to treat the whole patient, not just the disease.

* Practice gaps are represented by inadequate translation of knowledge into action.

## Data Availability

Not applicable.
